# Cell based assay identifies TLR2 and TLR4 stimulating impurities in Interferon beta

**DOI:** 10.1038/s41598-017-09981-w

**Published:** 2017-09-05

**Authors:** Lydia Asrat Haile, Swamy Kumar Polumuri, Roshni Rao, Logan Kelley-Baker, Dimitri Kryndushkin, Rajesh Rajaiah, Tomer Israely, V. Ashutosh Rao, Daniela Verthelyi

**Affiliations:** Laboratory of Immunology, Division of Biotechnology Review and Research III, Office of Biotechnology Products, Center for Drug Evaluation and Research, Food and Drug Administration, Silver Spring, Maryland United States of America

## Abstract

Immunogenicity can have devastating consequences on the safety and efficacy of therapeutic proteins. Therefore, evaluating and mitigating the risk of product immunogenicity is critical for the development these products. This study, showed that Betaseron and Extavia, which are reported to be more immunogenic among IFNβ products in clinical usage, contain residual innate immune response modulating impurities (IIRMIs) capable of activating NF-κB and induced expression of inflammatory mediators. These IIRMIs were undetectable in Rebif or Avonex. The stimulatory effect was attributed solely to IIRMIs because it was evident in murine cells lacking the interferon receptor (IFNAR). The IIRMIs in Betaseron and Extavia triggered NF-κB activation in HEK-293 cells bearing TLR2 and TLR4 in MyD88 dependent manner. Importantly, the IIRMIs in Betaseron induced up-regulation of IL-6, IL-1β, and ccl5 in the skin of IFNAR knock out mice following subcutaneous administration. This indicates that trace level IIRMIs in Betaseron could contribute to the higher immunogenicity rates seen in clinics. Together these data suggest that cell based assays can reveal subtle but clinically relevant differences in IIRMIs following manufacturing changes or between products with the same active ingredients but different manufacturing processes. Appreciating these differences may inform immunogenicity risk assessments.

## Introduction

Over the past decades, development of protein-based therapeutics has transformed the treatment paradigm and outcome of many chronic and serious diseases. Despite significant advances in recombinant protein engineering, a high incidence of unwanted immunogenicity is still encountered in clinical settings, resulting in important challenges to safety and efficacy^1^. Many factors have been reported that contribute to immunogenicity risk of therapeutic proteins^2–7^. Although for most products the downstream purification steps are designed to minimize impurities, some residual impurities can be present in the final drug product in trace levels that can be hard to detect and quantify. In recent years our group and others have shown that trace levels of process, host cell derived impurities, and contaminants introduced unintentionally during the manufacturing process can activate the receptors of innate immune cells increasing the risk of developing anti-drug antibodies (ADA)^8–11^. Some of those impurities, particularly those derived from adventitious microbial agents or resulting from cellular stress can be recognized by germline-encoded pattern recognition receptors (PRRs) that are expressed on barrier endothelia, antigen presenting cells (dendritic cells and macrophages) and various immune cells^12^. A growing number of PRR have been identified including Toll-like receptors (TLRs), C-type lectin receptors (CLRs) expressed predominantly on the cell surface, as well as NOD-like receptors (NLRs and several receptors that recognize nucleic acids in the cytoplasm)^12, 13^. Activation of PRRs by IIRMI can lead to transcriptional regulation of distinct genes and nuclear translocation of transcription factors depending on the type of receptors or cells^14^. This in turn, can lead to recruitment of immune cells to the site of the inoculation, activation and maturation of antigen presenting cells, inhibition of regulatory T cells, or direct activation of B cells, ultimately resulting in increased immunogenicity risk^15–17^.

While the adjuvant effect of PRR is well known, recent studies from our group showed that minute amounts of purified PRR agonists (PRRAg) can activate immune cells locally and systemically as evidenced by increased levels of immune related cytokines such as IFNγ, TNFα and IL-6^10, 18, 19^. *In vivo*, studies in non-human primates showed that the subcutaneous inoculation of minute amounts of PRRAgs induced the local expression of immune and inflammation related genes as well as increased ADA when these PRRAgs were co-injected with a therapeutic protein^20–22^. This indicated that very low levels of PRRAgs in a therapeutic product can increase the risk of immunogenicity.

Recent advances in product characterization have enabled sponsors and regulatory agencies to establish whether therapeutic proteins are highly similar, however there remain concerns that differences in the manufacturing processes can lead to subtle differences in impurities that could impact on the product’s immunogenicity risk^8, 23–25^. Despite the broad spectrum of IIRMIs that could be present in therapeutic proteins and peptides, current testing strategies are often limited to the use of the LAL test to measure endotoxin, a PCR test to detect host cell DNA, and ELISA based tests for host cell proteins. To broaden the spectrum of IIRMI tested in therapeutic proteins we recently established a test that uses human and mouse macrophage cell lines rich in PRRs and showed it can detect low levels of purified PRRAgs^26^. In the current study, we test the capacity of this platform to detect differences in IIRMIs among commercial therapeutic products produced on different manufacturing platforms using recombinant human interferon beta (IFNβ) as a model.

Interferon beta is an immunomodulatory cytokine used to treat multiple sclerosis. There are four RhIFNβ products marketed in US under different brand names: Rebif (IFNβ-1a), Avonex (IFNβ-1a), Betaseron (IFNβ-1b) and Extavia (IFNβ-1b) (Supplementary Table 1)^27^. RhIFNβ-1b products are produced in *E. coli* and are non-glycosylated (Supplementary Table 1)^28^. They also have an amino acid substitution at position 17 and lack the N-terminal Methionine^29^. Recombinant human IFNβ-1a products are produced in mammalian CHO cells, have an amino acid sequence identical to the human protein and are glycosylated. Multiple studies indicate a higher incidence of antibodies for patients treated with Betaseron and Extavia compared to those that receive Rebif and Avonex^30–32^. Importantly, the development of binding or neutralizing antibodies to these products correlate with a decline in bioavailability, increased frequency of relapses, and poorer clinical outcomes^32–35^.

In this study we hypothesized that differences in the manufacturing platform could result in differences in IIRMIs in the products that could impact on the immunogenicity risk of each product. Using the cell-based assay described above, we show that *E. coli* derived products contain TLR2 and TLR4 stimulating impurities and induce increased transcripts for pro-inflammatory genes including IL-1α, IL-1β, nos2 and cll5 both *in vitro* and *in vivo*. While we cannot establish whether these impurities contribute to the higher immunogenicity rate observed in patients treated with Betaseron and Extavia, the method was able to detect impurities in therapeutic proteins that were not in evidence through routine testing and show that the type of response they elicit could impact on the immunogenicity risk of the product. Understanding the IIRMI content in a product can help predict and control product immunogenicity reducing residual uncertainty and informing the need for clinical trials to assess product immunogenicity.

## Materials and Methods

### Reagents

Lots of IFNβ products were obtained from Betaseron (Bayer Health Care pharmaceuticals, Montville, NJ (lots# 33937A, 44036A, 52143A, 52044A), Extavia (Novartis Pharmaceuticals, East Hanover NJlots # 33930A, 44113-1A), Avonex (Biogen Inc., Cambridge MA lots # R01022, P32151), and Rebif (Serono Inc, Randolph, MA lots # AU013278, AU010438, AU015150, AU0146341). The presentation of Betaseron and Extavia products was as lyophilized powder containing 0.3 mg IFNβ-1b (9.6 MIU). The products were reconstituted on the day of use per manufacturer’s instructions using 1.2 mL of the supplied diluent. The Rebif® dosage form is 0.5-mL aqueous-solution formulation in a prefilled syringe containing 44-μg IFN-β-1a (24 MIU). Each lyophilized vial of Avonex^®^ contains 33 µg of interferon beta-1a. The powder was re-constituted with 1.1 mL of diluent provided by the manufacturer, so that the final concentration was 30 µg/ml (6 MIU). The prefilled syringe Avonex® dosage form contain 30 μg IFNβ-1a. All IFNβ products were stored and handled according to label instructions and used before expiry. All calculations for test product concentration were based on international units (IU) as labeled. The maximum concentration used in our *in vitro* assays was equivalent to 1/8th, 1/12th or 1/6th of the clinical dose for Betaseron/Extavia, Rebif and Avonex respectively. LPS from *Salmonella Minnesota* Re 595 was purchased from Calbiochem (Darmstadt, Germany). Pam3CSK4, FSL-1, were purchased from InvivoGen (San Diego, CA, USA) and used at the concentration indicated in each individual figure.

### Cell culture

#### RAW-Blue cells and HEK 293 h-TLR transfected cells

Murine RAW-Blue, HEK-BLUE-hTLR2 and HEK-BLUE-hTLR4 cells carrying a SEAP reporter construct were purchased from InvivoGen (San Diego, CA, USA). Cells were grown in DMEM supplemented with 10% FCS, 2 mM L-glutamine, 100 µg/mL Normocin in the presence of selection antibiotic and passaged when 70% confluence was reached per manufacturer’s recommendation. Cells were scraped and resuspended in test media as suggested by manufacturer for testing (InvivoGen, San Diego, CA, USA).

#### Testing of products on NF-κB reporter cells lines

Cells were plated at the density indicated on each figure legend in flat bottom 96-well plates in a 100 µL final volume and a serial dilution of respective products or TLR ligands were prepared and added at 100 µL volume as indicated in figure. In some experiments, Polymyxin B (InvivoGen) was used at the final concentration of 25 µg/mL in order to block endotoxin activity. In some experiments, IFNβ products were treated with proteinase K for 1 h at 37 °C and inactivated at 65 °C for 10 minutes to degrade protein impurities. After 24 h of stimulation, supernatants were collected and NF-κB activation was determined using detection medium QUANTI-Blue prepared according to manufacturer recommendations (InvivoGen, San Diego, CA, USA).

### Animals

C57BL/6 (B6), C57BL/6-IFNAR^−/−^ (IFNAR KO) and C57BL/6-MyD88^−/−^ (MyD88 KO) mice used in this study were bred as homozygous breeding pairs (>20 generations). Mice were housed in microisolator cages with a 12-hour day/night cycle and given food and water ad libitum in the specific pathogen-free, AAALAC accredited animal facility of the U.S. Food and Drug Administration’s Division of Veterinary Medicine (Silver Spring, MD). All animals and experimental protocols were reviewed and approved by the Food and Drug Administration Institutional Animal Care and Use Committee (FDA-IACUC) and performed accordingly.

### Splenocyte preparation

Mice were housed in microisolator cages with a 12-hour day/night cycle and given food and water ad libitum in the specific pathogen-free, AAALAC accredited animal facility of the U.S. Food and Drug Administration’s Division of Veterinary Medicine (Silver Spring, MD). All animals and experimental protocols were reviewed and approved by the Food and Drug Administration Institutional Animal Care and Use Committee (FDA-IACUC). All experimental animal protocols were performed according to the ethical principles of FDA-IACUC. 8–10 weeks old wild type C57B6, IFNARKO or MyD88 KO were sacrificed and spleens were removed. Spleens were mashed using the rubber end of plunger from 2 mL syringe. The single cell suspension was passed through 70-µm cell strainer. Splenocytes were suspended at 6 × 10^6^ cell/mL concentration. 500 µL of cell suspension were plated on 24 well plate and 500 µL of drug product or TLR ligand (positive control) was added at the concentration indicated on the figure legend.

### ***In vivo*** experiments

C57BL/6-IFNAR^−/−^ (IFNAR KO) mice were shaved one day prior to start of the experiment. Mice were injected with 1 × 10^6^ IU of Betaseron or Rebif in 50 µL subcutaneously (SC) using a 30 gauge needle. Control mice (n = 4) received 0.9% normal saline solution (Quality Biological, Gaithersburg, MD, USA). Skin biopsies were obtained from the site of injection under aseptic conditions 5 hours post-injection of IFNβ using disposable 4 mm biopsy punches (Miltex, York, PA, USA). The tissues were immediately submerged in TRIzol reagent (Invitrogen, Carlsbad, CA, USA), and stored at −80 °C until processing. To homogenize the tissues, skin biopsies were thawed and mixed with one millimeter-diameter Zirconia Beads (Biospec Products, Bartlesville, OK, USA). The samples were shredded by glass-bead friction using the Precellys 24, Cryolys system (Bertin Technologies, France) using the following settings: 6800 rpm; number of runs 2; run time 30 s; pause time 30 s. Homogenates were transferred into sterile 2 mL eppendorf tubes and total RNA was isolated as described below.

### qRT-PCR analysis and Taqman Low density array (TLDA)

Cytokine mRNA measurement following stimulation of cells and skin was performed by qRT-PCR. Total RNA was prepared from cells lysate using TRIzol (Invitrogen, Carlsbad, CA) as per manufacturer instructions. Contaminating genomic DNA was removed with TurboDNase (Ambion, Austin, TX). Subsequently RNA (1 μg/mL) was reverse transcribed into cDNA using high capacity cDNA Reverse Transcription Kit (Applied Biosystem, Foster City, CA) as per manufacturer recommendation. The qRT-PCR reactions were conducted in 1X Universal master mix (Applied Biosystem, Foster city, CA) with 1/20 volume cDNA/reaction for individual gene expression assays, in a Viia7 Real-time PCR system (Applied Biosystem, Foster city, CA). Custom-made human specific TaqMan® Micro Fluidic Cards containing panels of 96 gene expression assays were loaded with a sample volume of cDNA solution equivalent to 250 ng of the original RNA, mixed with 2x Universal Master Mix (ThermoFisher, Carlsbad, CA) The Ct values obtained for each gene were directly normalized to housekeeping values (GAPDH or 18S) in order to obtain ΔCt. Change in the expression levels of mRNA in stimulated cultures were indicated as fold increase over media treated cells using 2^−ΔΔCt^.

### Limulus amoebocyte lysate assay

The endotoxin content in IFNβ products were determined by using the endpoint or kinetic chromogenic LAL Kinetic-QCL assays according the manufacturers instruction (Lonza, Walkersville, MD).

### Aggregation of IFNβ products

Protein aggregates were made by mechanical stress by stirring 1.2 mL for Betaseron or Rebif using a Teflon stirrer in a glass vial for 20 hours at Room temperature (1100 rpm, 25 °C). The formation and size of protein aggregates was confirmed by visual observation, by measuring optical density at wavelength 200–500 nm with a spectrophotometer, and by micro-flow imaging (MFI) using a MFI500 from Proteinsimple (San Jose, CA). Oxidation of IFNβ-1a (Avonex without HSA) was performed by overnight treatment with 5 mM ascorbic acid, 5 mM H2O2 and 5 µM Cu (II) at 25 °C. Oxidants were removed by spin column filtration (7k Zeba™ Spin Desalting Columns, Thermo Fisher Scientific) applied twice. Aliquots of the original IFNβ-1a before oxidation and the oxidized IFNβ-1a were evaluated side-by-side using SDS-PAGE followed by silver staining (Pierce Silver Stain Kit, Thermo Fisher Scientific). An increase in total carbonylation in the oxidized IFNβ-1a was confirmed by the carbonyl ELISA^36^.

### Statistical analysis

Statistical significance was determined using “t” test or ANOVA with post-test multiple comparisons using GraphPad Prism 7.0. In all cases, a *P* value < 0.05 was considered significant. For multiple comparisons the stars in the graphs correspond to the post-test comparison with *p < 0.05, **p < 0.01, and ***p < 0.001.

## Results

### IFNβ products induce inflammatory genes in macrophages

 It is established that recombinant human IFNβ can induce binding and neutralizing antibodies that reduce their therapeutic efficacy. Among the marketed products, the immunogenicity rate appears to be higher for the IFNβ-1b products produced in *E. coli* than the IFNβ-1a produced in CHO cells and this has been generally ascribed to minor differences in sequence and presence of aggregates (Supplementary Table 1)^32, 37^. We hypothesized that these products could also differ in the content of trace levels of IIRMI, which may contribute to the difference in immunogenicity rates.

**Figure 1:**
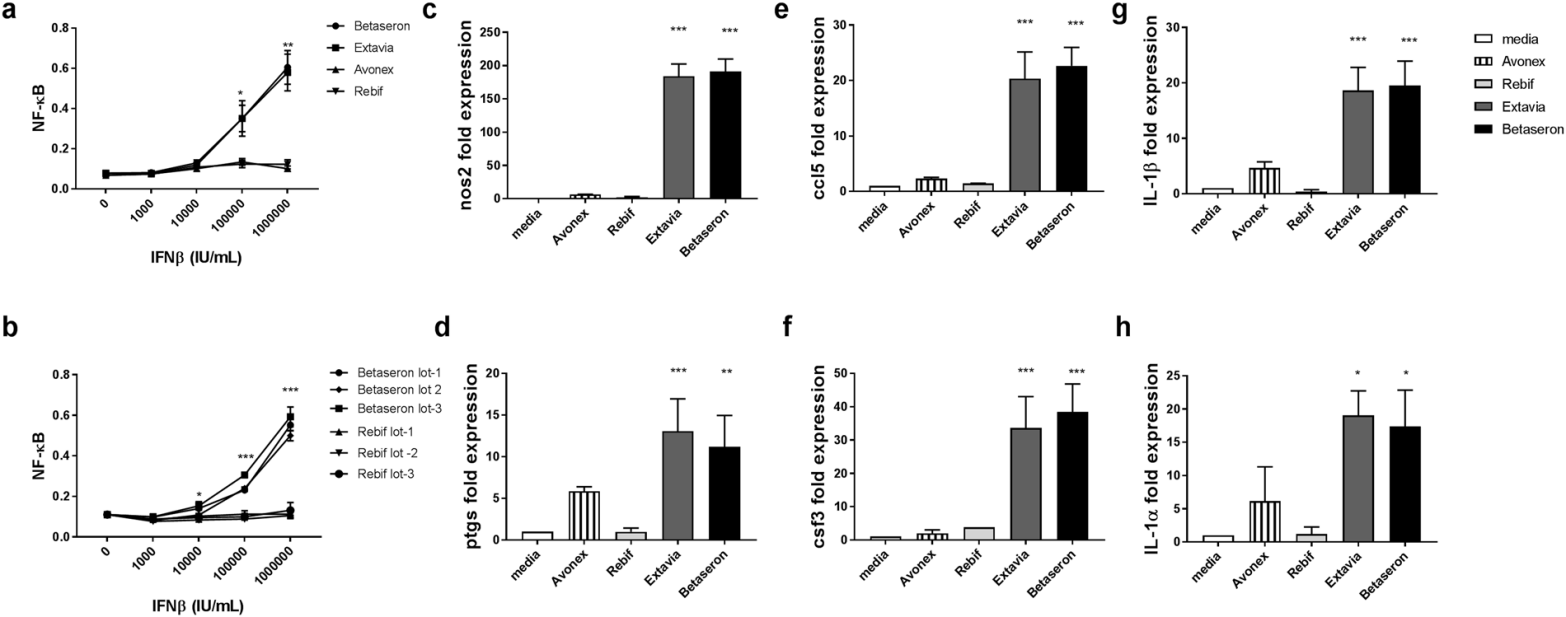
IFNβ products display a differential pattern of impurity related innate immune response gene expression. (**a**) RAW-Blue cells were treated with indicated concentration Betaseron, Extavia, Avonex and Rebif for 24 h. The level of NF-κB activation in the supernatant was measured using QUATI-Blue as described in materials and methods. Each point represents mean ± SD pooled from 2 independent experiments. (**b**) Three different lots of Betaseron and Rebif were tested on RAW-Blue cells as above (**c–h**) Transcript levels of nos2, ptgs, ccl5, csf3, Il-1b and Il-1a in RAW-Blue cells Fold expression is calculated over media treated cells, data shows mean ± SD derived from the 2–3 independent experiments. *p < 0.05, **p < 0.01, ***p < 0.001.

Currently testing of innate immune response modulating impurities (IIRMIs) in products is focused on endotoxin and nucleic acids, however, depending on the cell substrate, adventitious agents and manufacturing process employed, other IIRMIs could be present. Most human cell lines bearing PRRs also have receptors for interferons and thus could be activated by the IFNβ products masking the presence of impurities. To avoid this we selected RAW-Blue cells, a murine cell line that does not express receptors for human IFN. We recently showed that RAW-Blue cells expressing a secreted embryonic alkaline phosphatase (SEAP) reporter construct inducible by NF-κB could be used to detect trace levels of different IIRMI in therapeutic products^26^. As shown in Fig. 1a, Betaseron and Extavia induced a dose dependent activation of NF-κB in RAW-Blue cells that was absent in cells stimulated with Avonex or Rebif. The effect was evident at concentrations above 100,000 IU/mL. Testing of 3 lots of Rebif and Betaseron indicated that the difference was consistent as all the tested lots of Betaseron activate NF-κB whereas none of the lots of Rebif did (Fig. 1b). Next we determined whether the increased activation of NF-κB was associated with increased expression of genes linked to inflammation and innate immune activation. As shown in Fig. 1c–h, Betaseron and Extavia, but not Avonex or Rebif, induced relatively higher mRNA levels for inflammatory mediators nos2, ptgs, ccl5, csf3, IL-1α, IL-1β *in vitro*. Our data suggested that RAW- Blue cells detected the presence of IIRMIs in IFNβ products Betaseron and Extavia, which are expressed in *E. coli*, but not in the IFNβ products produced in CHO cells.

### Impurities in Betaseron and Extavia are responsible for the induction of inflammatory mediators

**Figure 2:**
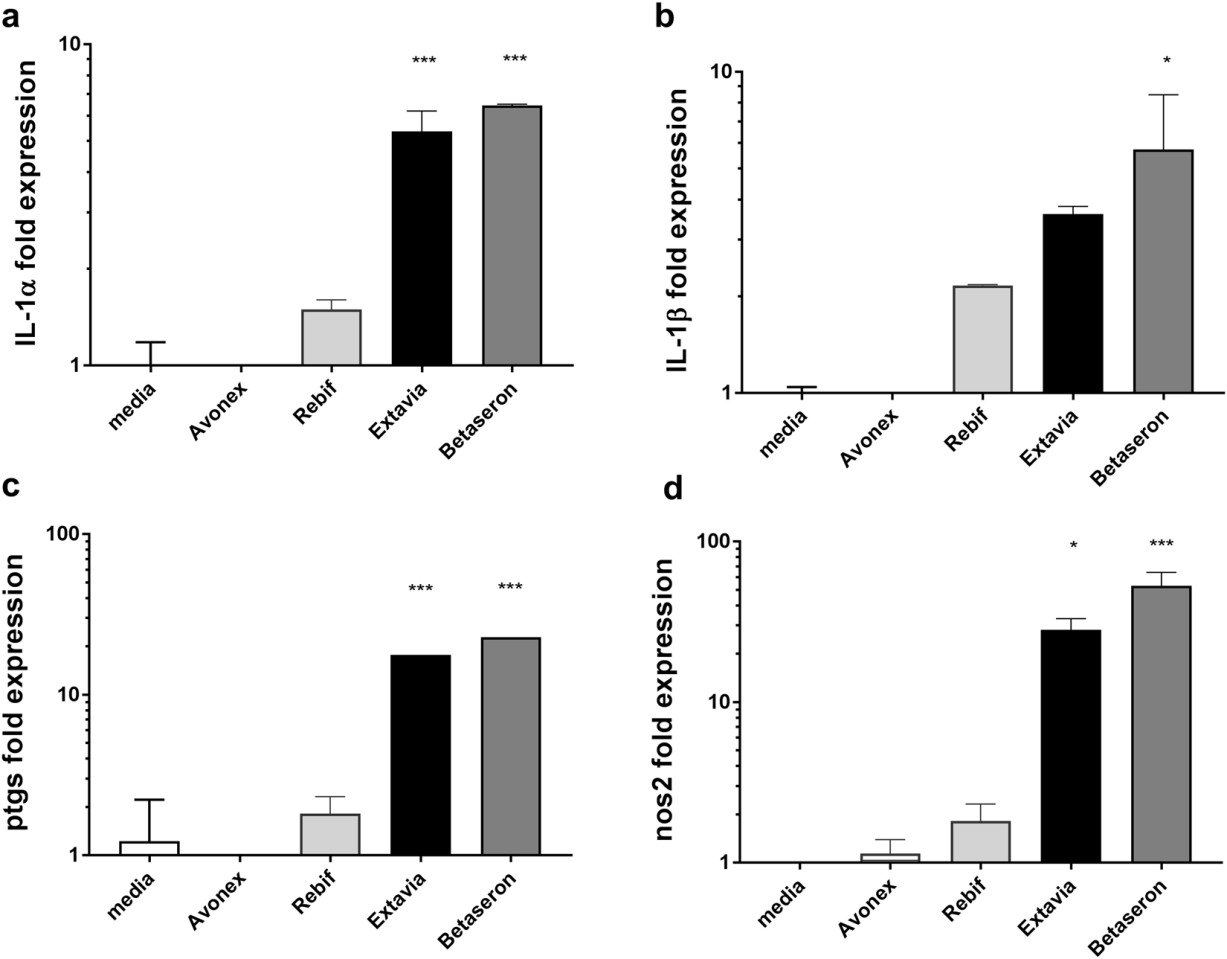
IFNβ is not responsible for the induction of inflammatory genes on mouse macrophage cell line. (**a–d**) Splenocytes from IFNAR knockout mice were stimulated with different IFNβ products for 24 h as described in Fig. 1. mRNA level of IL-1α, IL-1β, ptgs and nos2 were determined by qPCR. Data derived from mean ± SD from triplicate culture. *p < 0.05, **p < 0.01, ***p < 0.001.

IFNs are known to modulate the expression of many interferon inducible genes. Despite the low homology between human and murine IFNs, all four IFNβ products showed similar modest increases in the activation of interferon-β inducible genes such as Ccl2, Csf-1, Cxcl10, Cxcl11, Socs1, Socs2 and Stat1 in the murine RAW-Blue cells suggesting a low level of activation of the murine IFN receptor. To establish that the signal detected using the RAW-Blue cells was due to IIRMIs and not derived from differences in the active product ingredient (API), splenocytes from IFNAR knockout mice were stimulated with the four different IFNβ products for 24 h. As shown in Fig. 2a–d, Betaseron and Extavia induced increased expression of il-1α, il-1β, ptgs2 and nos2 relative to unstimulated splenocytes in the absence of IFNAR. Conversely, no increase in il-1α, il-1β, nos2 or ptgs was evident for the splenocytes incubated with similar levels of Avonex or Rebif. These results confirmed that *E. coli*-derived products have IIRMIs that induce an inflammatory response independent of the IFN- β activity.

### IIRMIs in Betaseron and Extavia signal through TLR2 and TLR4

**Figure 3:**
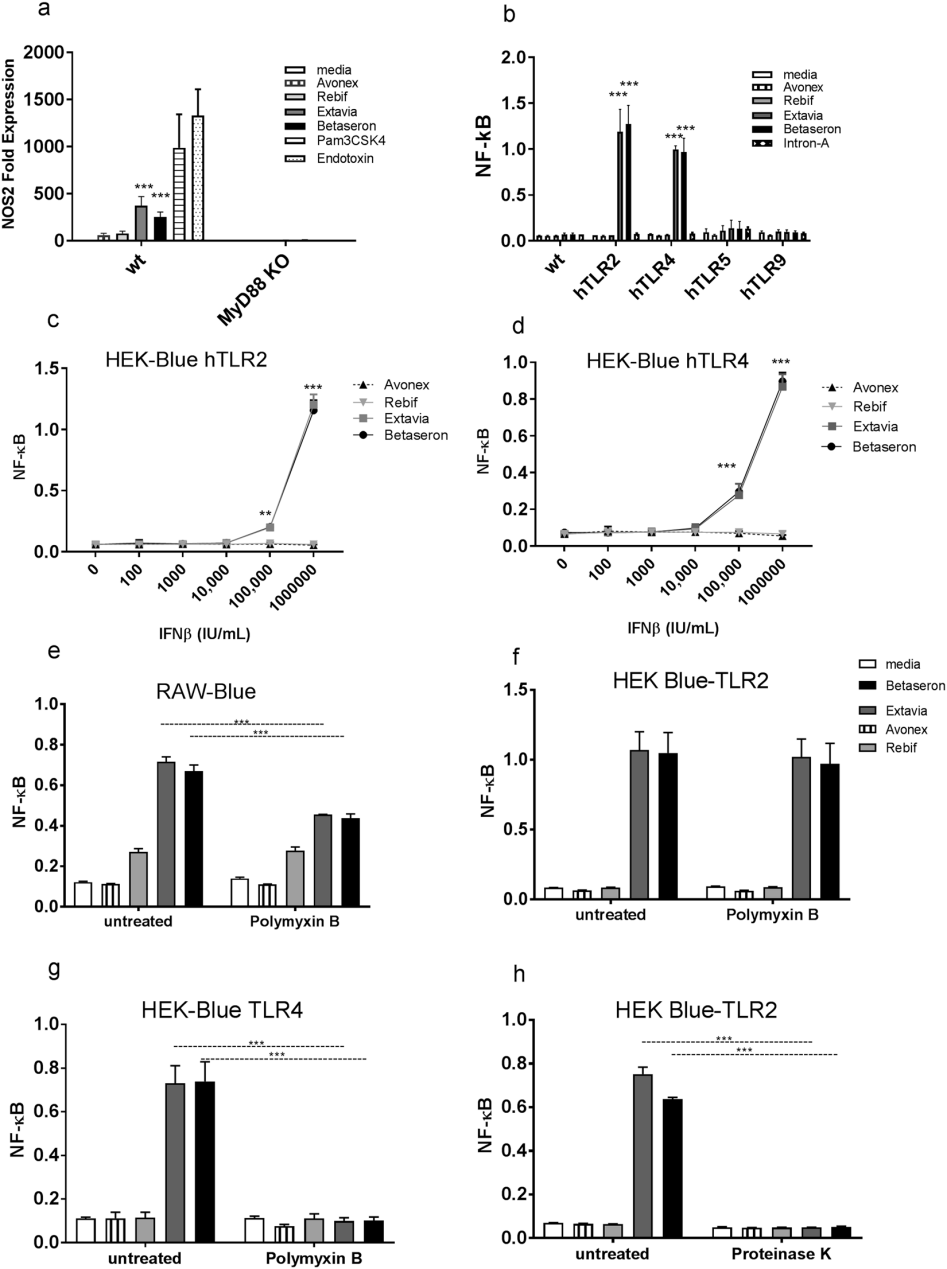
TLR2 and TLR4 activating IIRMIs are present in Betaseron and Extavia. (**a**) IFNβ products (1 × 10^6^ IU/mL) or positive control endotoxin/Pam3CSK4 (10 ng/mL) were added to splenocytes from Myd88 knock out mice for 24 h. The expression of nos2 was quantified by qPCR. The fold expression was calculated versus media-treated cells. (**b**) HEK-Blue-hTLR transfectants were treated with 1 × 10^6^ IU/mL of Avonex, Rebif, Extavia, Betaseron and Intron-A (Interferon alfa-2b). Immune response was measured by NF-κB activation. (**c,d**) A dose response analysis of IFNβ products at increasing concentration on HEK-Blue hTLR4 and HEK-Blue hTLR2 cell lines. The result shows mean ± SD from 2 different experiments. (**e**) Avonex, Rebif, Extavia and Betaseron were tested on RAW-Blue (**f**) HEK-Blue hTLR4 (**g**) HEK-Blue hTLR2 cells in the presence or absence of polymyxin B (25 μg/mL). A 24 h culture supernatants were tested for NF-κB activation. (**h**) IFNβ products were treated with Proteinase K for 1 h at 37 °C and added to HEK-Blue hTLR2 cells. Data shown is mean ± SD from two experiments run as triplicates. *p < 0.05, **p < 0.01, ***p < 0.001.

Toll like receptors are thought to be the main family of PRR that sense the presence of microbial and stress related signals. All of the TLR, except for TLR3, signal their activation using adaptor protein MyD88. To determine whether the potential IIRMIs in Betaseron and Extavia were signaling through TLRs we tested the gene expression induced by the four IFNβ products using splenocytes from MyD88 KO mice. Splenocytes from C57BL/6 wild type mice showed increased expression of nos2 when incubated with Extavia and Betaseron but not Rebif or Avonex. On the other hand, Extavia and Betaseron failed to induce nos2 (Fig. 3a) in splenocytes from MyD88 KO mice suggesting that the IIRMIs inducted inflammatory genes through the TLR system.

In order to identify the nature of the IIRMIs that activated NF-κB and inflammatory mediators in Betaseron and Extavia, we next tested the products on a panel of commercially available HEK-293 cells that are stably transfected with individual human TLRs and carry an NF-κB-inducible reporter gene (HEK-BLUE-hTLR). As shown in Fig. 3b, Betaseron and Extavia contain IIRMIs that signal through TLR2 and TLR4 but not TLR5 or TLR9. None of the TLR expressing cell lines was activated by treatment with Avonex or Rebif. Of note, a different product produced in *E. coli*, Intron A (Interferon alpha-2b) did not induce NF-κB activation (Fig. 3b). This suggests that not all products produced in *E. coli* contain residual IIRMIs that trigger TLR2 and TLR4 activation. The activation of HEK-BLUE hTLR2 and HEK-BLUE hTLR4 was dose dependent and required a minimum of 100,000 IU/mL Betaseron or Extavia to obtain detectable increases in expression relative to unstimulated cells (Fig. 3c,d). Together, our result showed that Betaseron and Extavia contain low levels of IIRMIs that activate an immune response through TLR2, TLR4 in a MyD88 dependent manner.

### Partial reduction of impurity mediated immune response by polymyxin B and proteinase K

TLR4 is known to be the receptor for endotoxin as well as for mannan, heat shock proteins, glycoinositolphospholipids and envelope proteins (RSV, MMTV), while TLR2 is known to respond to peptidoglycans from gram positive bacteria, as well as other microbial antigens such as porin, MALP-2, lipophosphoglycan, and zymosan^12, 38^. The presence of endotoxin in therapeutic products is often tested using the LAL test. Betaseron and Extavia activated TLR4 bearing cells suggesting they contained trace levels of endotoxin, however none the products showed evidence of endotoxin when tested using the LAL test (Supplementary Figure 1). Previous studies have suggested that the formulation of the product may reduce endotoxin recovery by LAL. To determine whether the *E. coli* derived IFNβ had trace levels of endotoxin that were not detected by the LAL assay we treated the interferon with Polymyxin B (PMB), a cyclic, cationic peptide antibiotic that neutralizes endotoxin. Addition of polymyxin B resulted in partial reduction of NF-κB activation by Betaseron and Extavia compared to untreated controls on RAW-Blue cells (p < 0.05; Fig. 3e). This suggested that the products contain trace levels of endotoxin that are not detectable using the LAL assay, and that endotoxin is not the sole contributor to the immune response observed in macrophages (Fig. 3e). Further analysis using HEK-BLUE TLR2 and HEK-BLUE hTLR4 cells showed that Polymyxin B completely abrogated the activation of the TLR4 bearing cells but not the activation of cells bearing TLR2 **(**Fig. 3f–g **)**. To further explore the discordance between the data obtained with the RAW-Blue cells and the LAL assay we spiked the products with known quantities of endotoxin and tested the samples using the LAL test. As shown in Supplementary Figure 1 the IFNβ products, except for Rebif, had reduced endotoxin recovery that could explain the discordance in the results. Further, our study showed that levels of HSA above 0.5%, consistent with those in the IFNβ formulation, interfered with the detection of endotoxin using the LAL assay (Supplementary Figure 1).

The studies above had shown that the addition of polymyxin B resulted in only a partial reduction in NF-κB activation in the RAW-Blue cells. Since previous studies^39^ had shown that contamination with endotoxin could result in the activation of TLR2 we next determined whether treatment with Polymixin B reduced the activation of HEK-BLUE TLR2 transfected cells. As shown in Fig. 3f, treatment of the products with polymyxin B did not reduce the NF-κB activation on TLR2 expressing cells indicating that a second impurity was present. However, treatment of Betaseron and Extavia with proteinase K, a serine protease that has broad cleavage specificity, abolished the response on HEK-BLUE TLR2 cells suggesting that the second IIRMI in the products is a protein (Fig. 3h).

#### Aggregation of IFNβ didn’t increase TLR2 and TLR4 response

**Figure 4:**
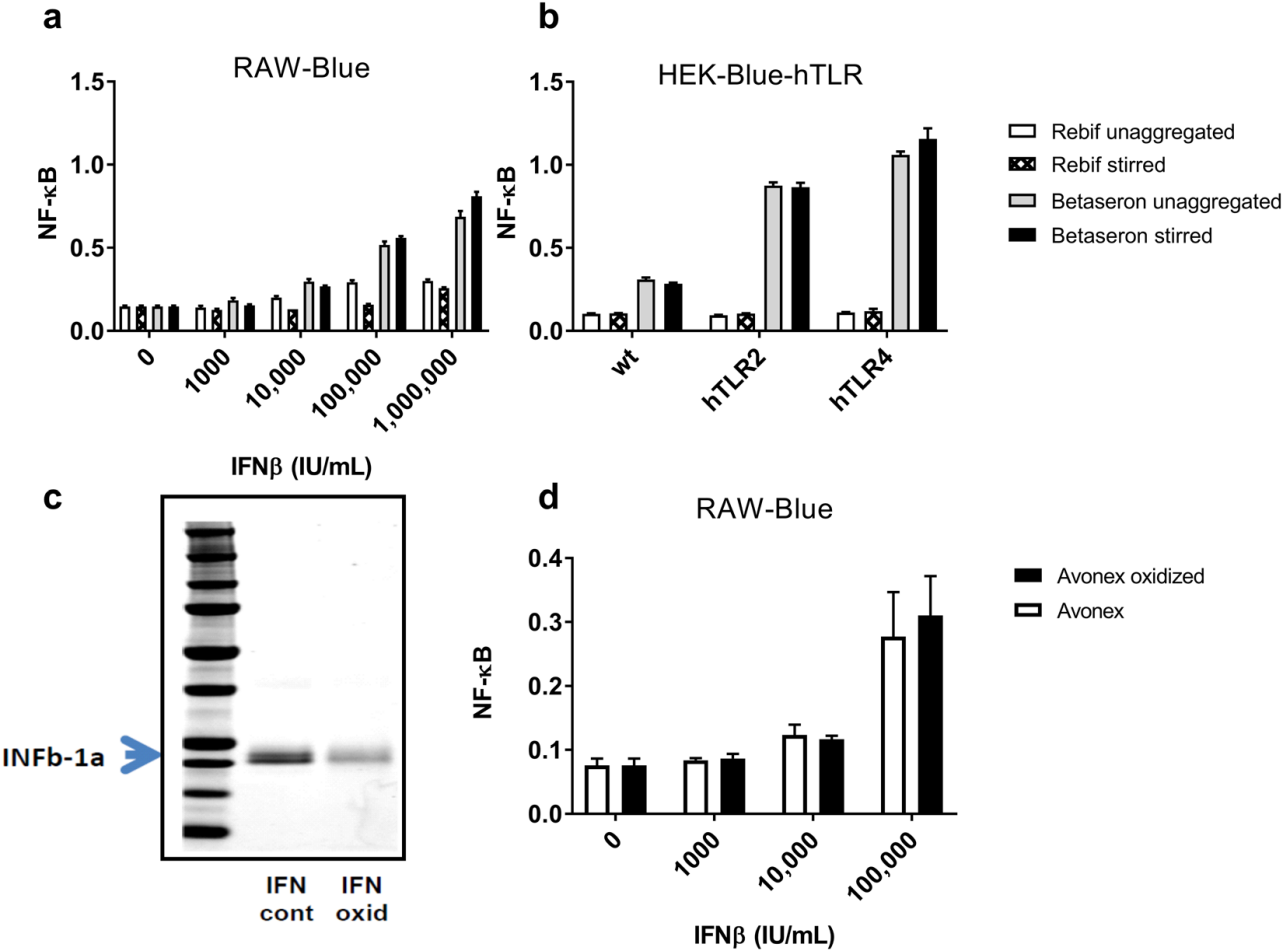
Aggregation of IFNβ didn’t increase TLR2 and TLR4 response. (**a,b**) Betaseron and Rebif were aggregated by stirring as described in M & M. RAW-Blue cells and HEK-Blue hTLR2 and HEK Blue hTLR4 transfected cell lines were cultured for 24 hours in the presence or absence of aggregated and unaggregated samples. (**c,d**) Aggregation of Avonex was induced by oxidation as described in materials and methods. Both the oxidized and un-oxidized products were tested for immune activation using RAW-Blue cells. Immune activation is measured by quantifying NF-κB from the 24 h culture supernatant colorimeterically. Data is mean ± SD from the triplicate culture.

Several studies have linked the higher immunogenicity risk of IFNβ−1b products particularly Betaseron to its propensity to form aggregates as compared to Avonex or Rebif^31, 40^. In order to explore whether differences in the levels of aggregates between products was responsible for the above observations Betaseron and Rebif were aggregated by stirring (Supplementary Figure 2) and then tested using RAW-Blue and HEK-BLUE-hTLR transfected cell lines. As can be seen in Fig. 4a,b aggregation of both Betaseron and Rebif didn’t modify the level of NF-κB activation observed in RAW-Blue, or in hTLR2 and hTLR4 transfected HEK-Blue cells as compared to un-aggregated drug products. Similarly, severe metal-catalyzed oxidation of Avonex resulted in protein degradation, and degradation products formed a poorly visible smear below and above the full-length protein band (Fig. 4c). Nevertheless, the oxidized IFNβ-1a did not modify the level of NF-κB activation (Fig. 4d). This provided additional evidence that the observed innate immune activation was driven not by the IFNβ proteins, but rather by IIRMI present in the INFβ products.

### Low levels of TLR 2 and 4 agonists replicate the increase in pro-inflammatory gene expression evidenced in Betaseron treated cells

**Figure 5:**
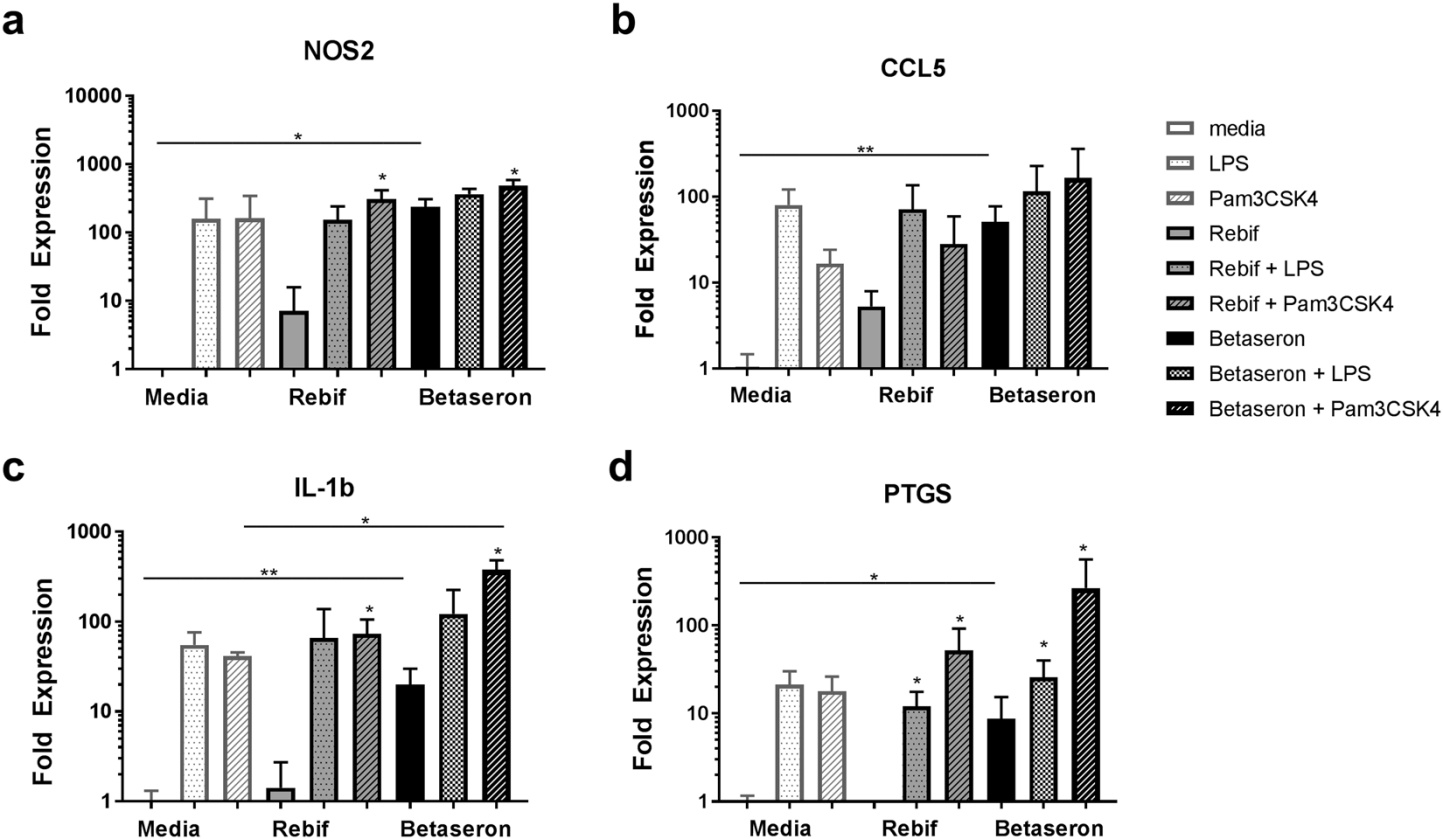
Low levels of TLR 2 and 4 agonists replicate the increase in pro-inflammatory gene expression evidenced in Betaseron treated cells. Transcript expression levels of selected innate immune response related genes (**a**) nos2, (**b**) ccl5, (**c**) IL-1β and (**d**) ptgs2 in RAW-Blue cells treated with Betaseron and Rebif or spiked with 1ng Endotoxin and 10ng of Pam3CSK4. Data shown is mean ± SD from two experiments run as triplicates. Statistical differences determined by ANOVA (identified by a line) or student t tests as appropriate; *p < 0.05, **p < 0.01.

To further determine whether low levels of impurities that triggered TLR could induce the expression of pro-inflammatory genes in cells cultured in the presence of Betaseron we next spiked Rebif with low levels of endotoxin or Pam3CSK4 (a purified TLR2 agonist). The expression of nos2, il-1β, ccl5, and ptpgs2, was used as markers of inflammation. As above, Rebif induced very low levels of mRNA for pro-inflammatory genes as compared to Betaseron in mouse derived RAW-Blue cells (p < 0.05). Spiking Rebif with endotoxin or Pam3CSK4 (Fig. 5 and Supplementary Table 2) led to an increase in the expression of inflammation-related genes by RAW-Blue cells that was similar to the one induced by Betaseron, but the level of activation was not different from that induced by TLR2 and TLR4 agonists alone indicating once again that Rebif did not have IIRMI (Fig. 5a–d). Similar spiking on cells cultured with Betaseron resulted in further increase in pro-inflammatory gene expression.

### Betaseron induce inflammatory genes *in vivo* on the skin

**Figure 6:**
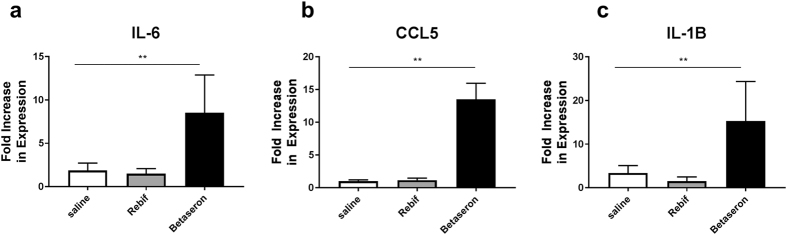
IIRMIs in Betaseron Induces inflammatory response by *in vivo* and response. (**a–c**) IFNAR knockout mice (n = 4–6/group) were injected with 1 × 10^6^ IU of Rebif, Betaseron or saline in 50 μL volume SC. After 5 h, mRNA levels of il-6, il-1β and ccl5 were quantified from skin lysates. Data derived from mean ± SD from two independent experiments. Statistical differences as determined by Bartlett’s ANOVA **p < 0.01.

The *in vitro* induction of innate immune related genes suggests that there are impurities in the product that could activate immune cells but does not necessarily imply that they would be sufficient to induce a response *in vivo* that could foster a change in the local milieu where the product is administered. To explore whether the impurities could lead to local immune activation we administered 1 × 10^6^ IU of Betaseron and Rebif subcutaneously to IFNAR knock mice and collected the local skin after 6 hours. Analysis of local gene expression showed an increase in local expression of IL-6, IL-1β and ccl5 mRNA in the skin *of* IFNAR knock out mice that received Betaseron, but not in the ones that received Rebif as compared with mice that received saline. This suggests that the levels of impurities in the product, while low, could foster a local inflammatory response that could facilitate antigen uptake by antigen presenting cells and product immunogenicity (Fig. 6a–c).

## Discussion

Immunogenicity risk cannot be predicted from protein structure alone as it is influenced by a myriad of often interacting parameters such as preexisting tolerance, immunological status and HLA type, underlying disease and presence of product aggregates^41^. One of the factors thought to impact product immunogenicity is the presence of product and process related innate immune response modifying impurities (IIRMIs) such as residual host DNA, host cell proteins, endotoxins and other microbial fragments, which may activate the local immune system where the product is administered, acting as inadvertent adjuvants. The range of impurities that could be present in the final drug product are likely to be largely dependent on the manufacturing, purification and fill-finish processes including the cell substrate platform and thus, changes in the manufacturing process can impact on the range and levels of IIRMI. Detecting the presence of such IIRMI is necessary for controlling the immunogenicity risk of therapeutic proteins and peptides. In these studies we sought to investigate whether a recently developed cell based assay could be used to detect impurities in commercial therapeutic proteins. Our studies showed that the method exclusively detected low levels of IIRMIs in Betaseron and Extavia that were not evident using the LAL test. IIRMIs in Betaseron and Extavia included low levels of endotoxin as well as at least one other protein IIRMI that activated TLR2 and they induced the expression of pro-inflammatory genes in a MyD88 dependent manner. Lastly, we showed that the levels of impurities that were present in the product were sufficient to induce local expression of cytokines in the skin of mice when injected subcutaneously suggesting that these IIRMI could contribute to an inflammatory milieu and activation of innate immune response in the space where the product is inoculated. Together these finding raise the possibility that the difference in IIRMI could be associated with the increased immunogenicity of the IFNβ-1b products and support the use of IIRMI characterization in the assessment of immunogenicity risk.

The assay system used to detect IIRMIs is based on the premise that macrophage cell lines bear numerous PRR and are able to detect a variety of impurities that could modify the milieu where the product is deposited. In a previous study we suggested that the combined use of multiple cell lines of mouse and human origin provided a broader set of highly sensitive PRR^26^ and this was particularly important since IIRMI can be complex and trigger multiple innate immune receptors. In this study the differences in IIRMI were evident when using the RAW-Blue cells but not when using the human MM6 cell line (Supplementary Figure 3). This was not surprising as the MM6 cell line has functional receptors for IFNβ, and therefore treatment of the cells resulted in the robust activation of multiple genes by the API including many of those that would be activated by the presence of IIRMI. Moreover, since IFN is known to modulate inflammation, it is possible that IFNβ directly inhibited the expression of inflammatory genes^42^. In contrast, given that the homology between human and mouse IFNβ is low (~60%), the response of the murine RAW-Blue cell line to the IFNβ was very modest, allowing the differences in IIRMI to surface. Similarly, in primary cells derived from IFNAR KO mice, which lack the capacity to respond to IFN, the effect of the IIRMI was clearly evident. This suggests that, the selection of cell lines to assess IIRMI should take in consideration the mechanism of action of the product, particularly when the API is immunomodulatory.

Our studies showing the activation of HEK293 bearing TLR4 and the corresponding partial reduction when the product was treated with Polymixin B strongly suggests that the product contains trace levels of endotoxin despite the negative finding using the LAL test. The LAL test is broadly used for testing endotoxin in therapeutic products^43^, however several conditions are known to reduce endotoxin recovery including the presence of surfactants, deoxycholate, or other formulation components that may alter endotoxin structure^44, 45^. As shown in Supplementary Figure 2, the ability of the LAL assay to detect endotoxin in the rhIFNβ products appears to correlate inversely with the albumin content. Previous studies examining the testing of endotoxin in human serum albumin have reported different outcomes when using the LAL test^46–48^.

Further investigation will be needed to assess whether the albumin in IFNβs can mask the presence of endotoxin in therapeutic proteins. Importantly, and not all products produced in *E. coli* induced the transcription of inflammatory genes as observed when Intron A was used.

Inoculation of IFNAR KO mice allowed us to establish that the IIRMI in a single dose of Betaseron but not Rebif resulted in induction of inflammatory genes IL-6, IL-1β and ccl5 in the skin. This is line with data from primate studies showing that trace levels of IIRMI activated the innate immune system in the skin milieu. However, our data didn’t show whether the expression of inflammatory genes at the injection site will translate into a clinically significant increase in immunogenicity and cannot be used to make quantitative predictions in humans.

Recombinant huIFNβ has been indicated for the treatment of relapsing and remitting multiple sclerosis (MS) since 1993, and reports regarding its immunogenicity and the reduction in efficacy that follows have been published since it was licensed^49^. Nabs appear usually within two years of treatment and can cross react with other IFNβ products. Indeed subjects are often asked to wait 2–3 months when switching between products to allow for the immunoglobulin response to subside^50, 51^. The immunogenicity of rhuIFNβ-1b products has been ascribed to multiple factors including the absence of glycosylation, the N terminal methionine, a substitution of a cystein for a serine in position 17^31, 52^ and aggregates. Of note, the rhuIFNβ-1a products, which are more homologous to the endogenous IFN, are also immunogenic albeit to a lesser degree^32^. Several studies suggested that the difference was possibly due to the difference in aggregate and particle content between the products^40^. Betaseron-rhIFNβ-1b has been shown to contain a higher percentage of large, soluble protein aggregates whereas size-exclusion chromatography indicates less than 2% aggregates in Avonex-rhIFNβ-1a^53, 54^. Further, dissociation of rhIFNβ-1b aggregates using high hydrostatic pressure considerably reduced immunogenicity in transgenic, immune tolerant mice supporting a role for product aggregates^55^. Recently, it was suggested that aggregates could activate innate immune cells partly by triggering TLR2 and TLR4 receptors^56^. Interestingly, in our model, submitting Betaseron and Rebif to physical or chemical destabilizing and aggregating conditions did not modify the readout of innate immune activation induced in the reporter cell line. This, together with the reduction in NF-κB activation in Polymyxin B treated product further suggests that the immune activation detected by the RAW-Blue as well as TLR2 and TLR4 transfected cell lines cells is due to IRMIs and not secondary to the presence of aggregates.

Over the last 15 years the recognition of the risk posed by product immunogenicity to safety and efficacy of therapeutic proteins and the inability to predict what products will induce clinically relevant immune responses has resulted in the requirement of extensive clinical trials during clinical development or as a post approval commitment. Recent improvements in analytical chemistry and product characterization are enabling new regulatory paths for peptides and proteins that could require fewer (351(k), 505(b)(2)) or no clinical studies (505(j); however concerns over potential immunogenicity remain. This is partly due to uncertainties regarding possible differences in product and process related impurities between products. At this time differences in techniques and lack of common controls hinder the determination of absolute levels of IIRMI that could be considered as increasing the risk of product immunogenicity. However the expectation is that technical progress, improved characterization and the correlation of the product attributes with clinical outcome will allow us eventually to make better predictions regarding the immunogenicity risk of product impurities. These studies demonstrate a possible approach to test protein products for the presence of IIRMI that may be used to inform an assessment of immunogenicity risk. The consequent reduction in residual uncertainty may facilitate review of these products and assist in the determination of the extent of clinical trials needed.

## Electronic supplementary material

Supplementary information
