# Supplementary material for: Cell based assay identifies TLR2 and TLR4 stimulating impurities in Interferon beta
Source: Sci Rep. 2017 Sep 5;7:10490. doi: 10.1038/s41598-017-09981-w (PMC5585229; doi:10.1038/s41598-017-09981-w)
Supplement: Supplementary file 1 — Supplementary information [file 41598_2017_9981_MOESM1_ESM.pdf]

Cell based assay identifies TLR2 and TLR4 stimulating impurities in Interferon beta

Lydia A Haile, Swamy Kumar Polumuri, Roshni Rao, Logan Kelley-Baker, Dimitri Kryndushkin, Rajesh Rajaiah, Israely Tomer, V. Ashutosh Rao, Daniela Verthelyi*

Supplementary Table 1. Formulation characteristics and immunogenicity profile of IFNβ products from different manufacturers used in the current study

Parameters	Rebif (IFNβ-1a)	Avonex (IFNβ-1a)	Avonex (IFNβ-1a)	Betaseron (IFNβ-1b)	Extavia (IFNβ-1b)
Produced in	CHO	CHO	CHO	E.Coli	E.coli
Glycosylation	Yes	Yes	Yes	no	no
Activity (10^6/mg)	300	300	300	32	32
pH of formulation	3.8	7.2	4.8	7.2	7.2
Stabilizer	HSA/ mannitol	HSA	Arginine HCl	HSA	HSA
Dosage form	Prefilled syringe	Prefilled syringe	Prefilled syringe	Prefilled syringe	Prefilled syringe
Buffer	acetate	phosphate	acetate	phosphate	phosphate
Injection site reaction	66%	4%	5%	85%	78%
NAB	24%	5%	5%	45%	45%
Manufactured by	Serono Inc.	Biogen	Biogen	Bayer	Novartis

Supplementary Table 2. Spiking Rebif with trace levels of *TLR 2 and 4 agonists replicate the increase in nos2 observed in Betaseron treated RAW-Blue cells.*

Condition	Media	Rebif	Betaseron
Media	1	1.1	191.6
Endotoxin (0.1ng/ml)	4.8	9.1	195.8
Endotoxin (1ng/ml)	50.5	93.3	316.3
Pam3CSK4 (1ng/ml)	34.6	66.6	420.1
Pam3CSK4 (10ng/ml)	146.4	230.28	1338.8

Transcript expression levels of nos2 in RAW-Blue cells treated with Betaseron and Rebif or spiked with 1ng Endotoxin and 10ng of Pam3CSK4. Data shown is mean ± SD from two experiments run as triplicates. Statistical differences determined by ANOVA (identified by a line) or student t tests as appropriate; *$p<0.05$, **$p<0.01$.

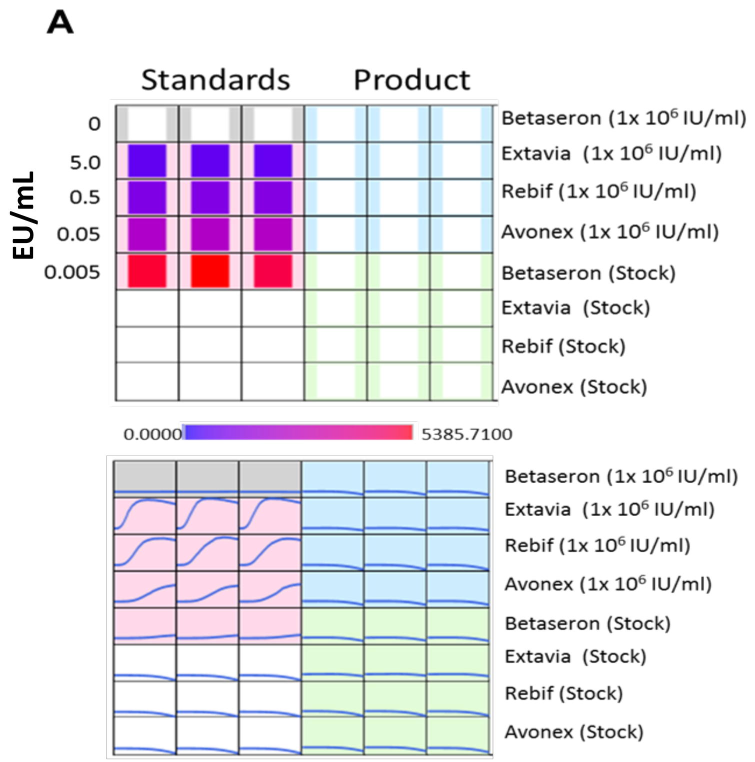

B

Products	% HSA
Betaseron	1.08%
Extavia	1.08%
Avonex	1.5%
Rebif	0.8%

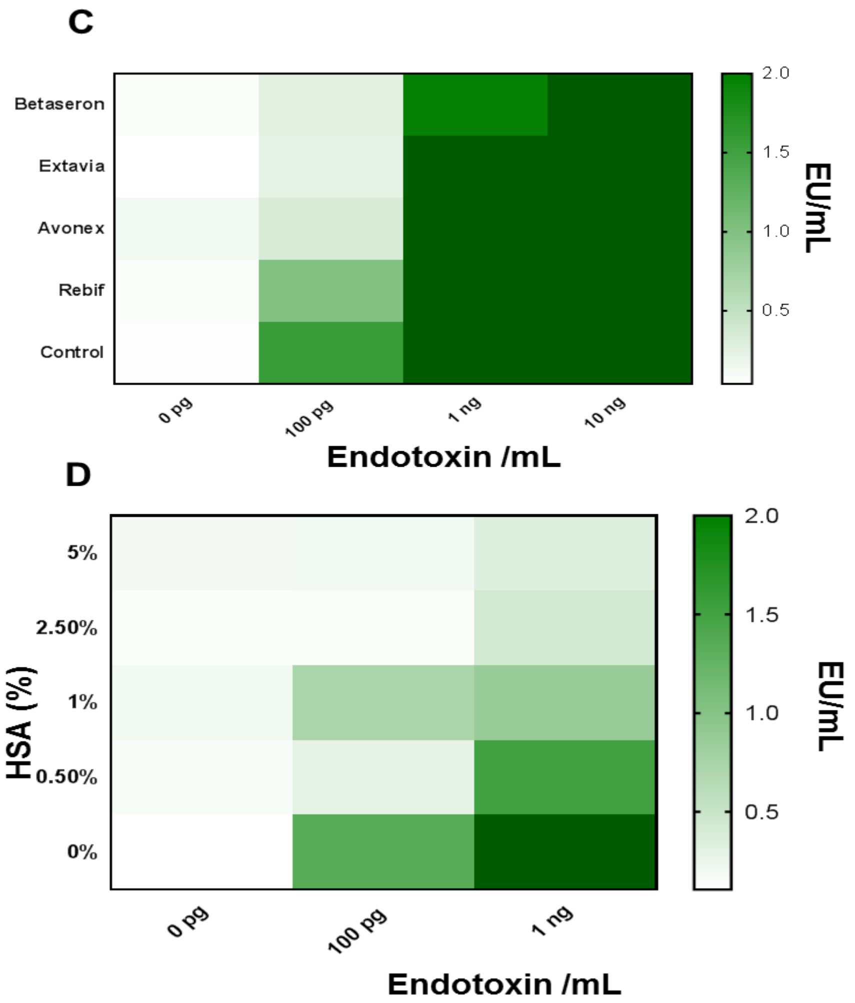

Supplementary Figure S1. Endotoxin levels as detected by LAL: potential impact of the HSA content. (A) IFNbeta products were tested using the Kinetic LAL testing system (Lonza). The sclae shows reaction time (in seconds). Similar results were evident when using the endpoint assay. (B) Human serum albumin content per label. (C) Products were spiked with different levels of endotoxin and tested by LAL assay to assess endotoxin recovery. (D) Different concentrations of HSA were prepared and spiked with endotoxin. Endotoxin levels were tested by endpoint LAL.

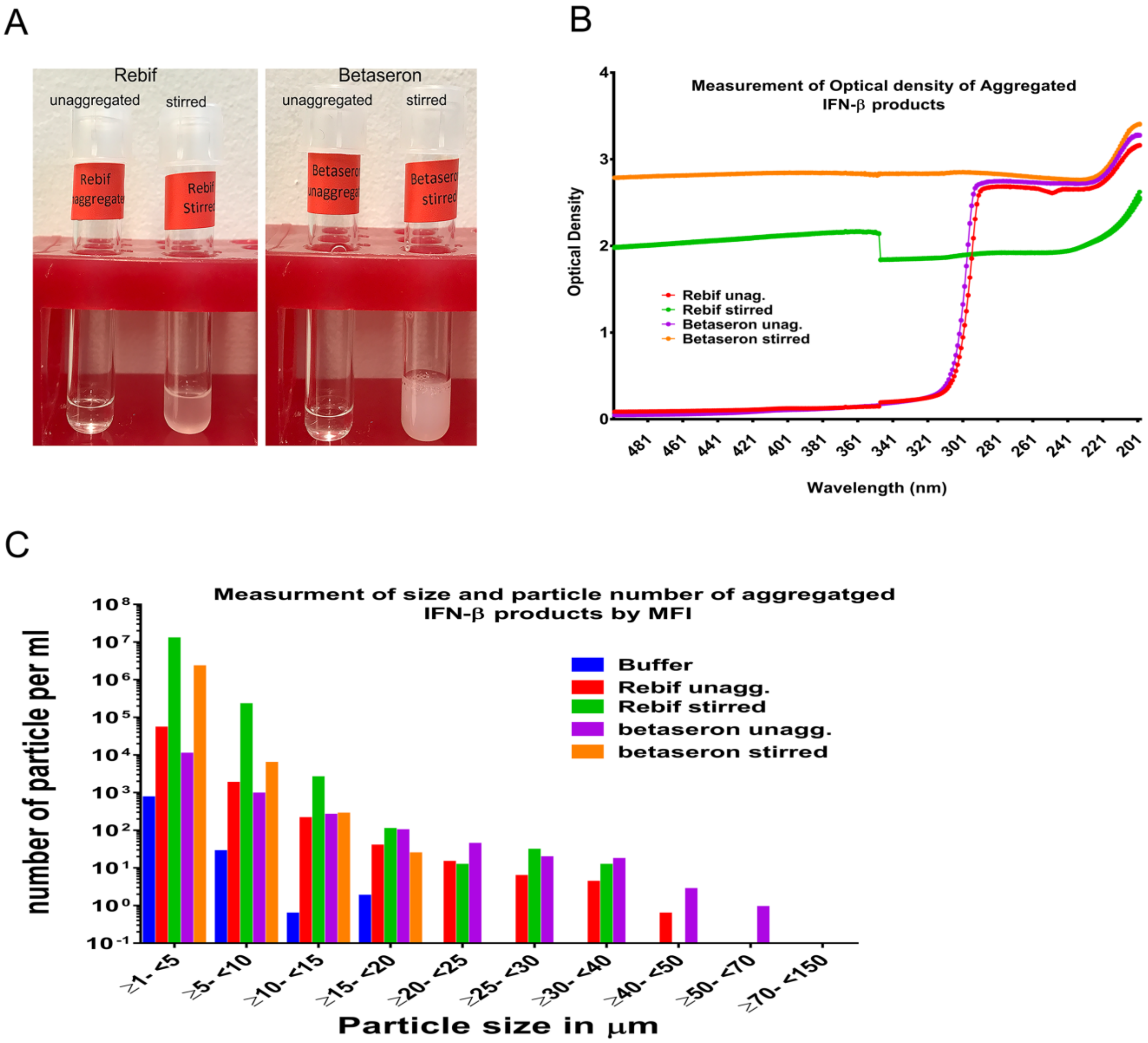

Supplementary Figure 2: Characterization of aggregates in IFNβ products. Rebif and Betaseron were stressed under stirred conditions with Teflon stirrer (1100 rpm) at Room Temperature. **(**A) picture showed turbidity following aggregation by visual observation. (B) The optical density of aggregated protein or unaggregated IFNβ was measured by using Carry 100UV.VIS spectrophotometer at wavelength 200-500 nm (C) Particle sizes and number of particle of IFNβ aggregates were analyzed by MFI

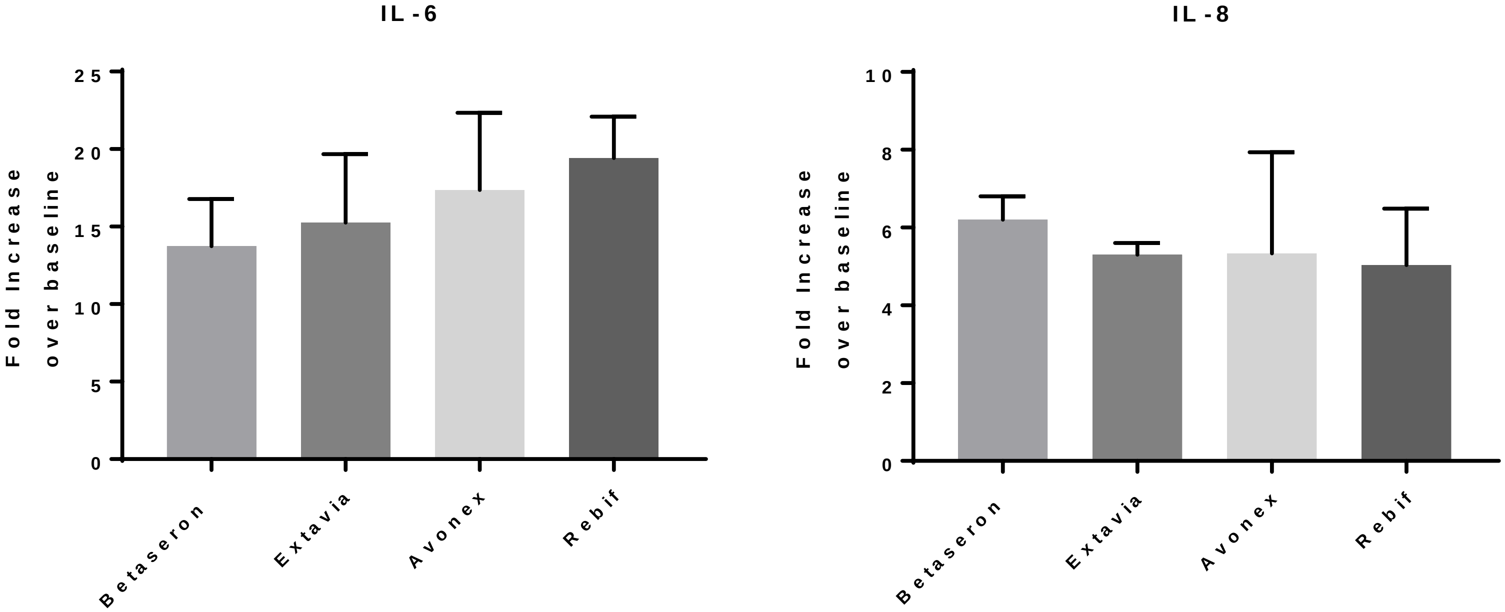

Supp. Figure 3. Comparison of IFNβ products on human macrophage cell line (MM6). MM6 cells were treated with $1x10^6$ IU/mL of different IFNβ products as shown in the figure. The level of mRNA for IL-6 and IL-8 were determined by qPCR. Data derived from triplicate samples derived from 3 different experiments.
